# Novel Technique to Repair Unilateral Cleft Lip: Separated Multiple Y-to-V-Plasty under Magnification

**DOI:** 10.29252/wjps.8.2.213

**Published:** 2019-05

**Authors:** Mohammad Ali Hoghoughi, Raha Habibagahi

**Affiliations:** 1 Department of Burn and Wound Healing Research Center, Plastic and Reconstructive Surgery Ward, Shiraz University of Medical Science, Shiraz, Iran;; 2 Department of Biomaterials, Orthodontic Research Center, School of Dentistry, Shiraz University of Medical Sciences, Shiraz, Iran

**Keywords:** Cleft lip, Y-to-V-plasty, Repair, Aesthetic

## Abstract

**BACKGROUND:**

Various techniques have been used for cleft repair such as the straight-line closure, the rotation advancement technique and the anatomic subunit approach which are famous new approaches gained popularity. However, these methods have several advantages and disadvantages and sometimes are difficult to adopt. In this study, we described our novel technique, known as separated multiple Y-to-V-plasty, in treatment of several cases of unilateral cleft lip.

**METHODS:**

Plastic surgeons usually try to avoid straight closure of the wound, especially in areas where they need to stretch, move and enhance the length of the wound in some stages of the procedure. Since the lip is a dynamic and active structure and is constantly moving, the use of straight-line incision and closure in that area is in conflict with this basic concept.

**RESULTS:**

In our newly introduced technique, we avoided a straight-line closure along the skin and mucosa for the repair of the unilateral cleft lip. This issue is of utmost importance in cases with severe loss of lip height and discrepancy.

**CONCLUSION:**

To acquire a natural and balanced shape in unilateral cleft lip repair, we recommended the novel Y-to-V-plasty technique as an effective method for severe unilateral cleft lip with enormous discrepancy.

## INTRODUCTION

Cleft lip, alveolus and palate comprise the most prevalent congenital facial defects. Repair of the unilateral cleft lip is a very rewarding and challenging procedure for plastic surgeons. Many clinicians prefer to carry out such surgeries during infancy, at 3-6 months of age.^1^ Currently, various surgical techniques are used for the repair of unilateral cleft lip defects. Straight-line closure technique for the repair of unilateral defects was introduced in 1840s.^2^ Since then, various techniques have been implemented for such procedures.

The most important surgical technique is Millard’s “rotational advancement” technique which is considered a basic technique for the repair of unilateral cleft lip.^3^ Other clinicians have greatly modified the original rotational advancement technique by making changes in the technique, including vermilion flaps^4^ and triangular advancement flaps.^5^^,^^6^ Other modifications of this technique include Mulliken’s modification^7 ^and Fisher’s^8^ recent anatomic subunit approximation technique. Designs made by cleft surgeons indicate attempts to solve vertical height deficiency of the lip, to balance the Cupid’s bow and to cause less disruption in the continuity of philtral column. Recent aims include symmetry and rearrangement of the cleft side nostril.^9^

In the majority of techniques used to date, in addition to the hard design, sufficient lip height at the cleft side cannot be achieved, especially in cases with severe discrepancy, and these methods result in some complications such as short lip, white skin roll discontinuity and mismatch, vermilion notching, failure to balance cupid’s bow and finally, nasal asymmetry which are usually repaired in a secondary procedure.^10^^-^^12^ Currently, plastic surgeons try to avoid straight closure of the wound, especially in areas, where they need to stretch, move and enhance the length of the wound in some stages of the procedure.^9^^,^^10^

In addition, there is general consensus that all straight-line scars can be shortened over time. Therefore, surgeons use methods such as stair-step incision in rhinoplasty through the columella, W-plasty, V-to-Y-plasty and Z-plasty. A large number of webs and contractures in joints, neck and movable areas have been treated with the use of these techniques in order to increase the length of the wounds and improve the aesthetic outcomes.^10^^-^^12^ In our newly introduced technique under the title “separate Y-to-V unilateral cleft lip repair under magnification”, the procedure is based on the comprehensive principle, since the lip is a dynamic and active structure and is constantly moving, the use of straight-line incision and closure in that area is in conflict with this basic concept. 

In all of the previous techniques,^3^^,^^7^^,^^8^ more than half the length of the wound has been straight-line closure in most cases. Z-plasty has been used above white skin roll and dry mucosa has increased the length of the scar and decreased the straight-line tension. Success in cleft lip repair is equally dependent on the repair design and technical skills. In order to repair unilateral cleft lip repair, CPHR, CPHL and CPHL’ points (Noordhoft’s points)^4^ are determined according to the general methods used in previous techniques.^3^^,^^7^^,^^8^ The third arms of the lower and last Y flap in the upper part of the vermillion should match the famous triangular flap as previously described by Fisher and others.^13^^,^^14^

There are advantages of using a microscope according to conventional methods. (i) There is no need to use epinephrine-lidocaine solution for homeostasis before surgery; as a result, hemostasis is achieved under microscope magnification using a bipolar cautery; (ii) Muscle dissection is performed with extreme precision, similar to that used before for dissection of levator veli palatini during cleft palate repair;^15^ and (iii) It is possible to prepare a delicate and precise Y design on nonphiltral and philtral sides under a microscope with magnification. This study has further customized the previous techniques and presented the experiences with the surgical management of the unilateral cleft lip and nasal deformity. 

## MATERIALS AND METHODS

This study described the modifications and discussed the surgical principles utilized in the unilateral cleft lip repair treating more than 20 patients with this method within 6 months of follow-up. Our new Y-to-V-plasty technique along skin and mucosa, particularly in severe discrepancies, resulted in a decrease in tension from the closure line and in an increase in the length of the wound in comparison with all the conventional unilateral cleft lip repair methods. In this method, the effect that we call an “accordion effect” probably reduced the wound tension in the Cupid’s bow, nostrils, alar base and columella on the cleft side.

**Fig. 1:**
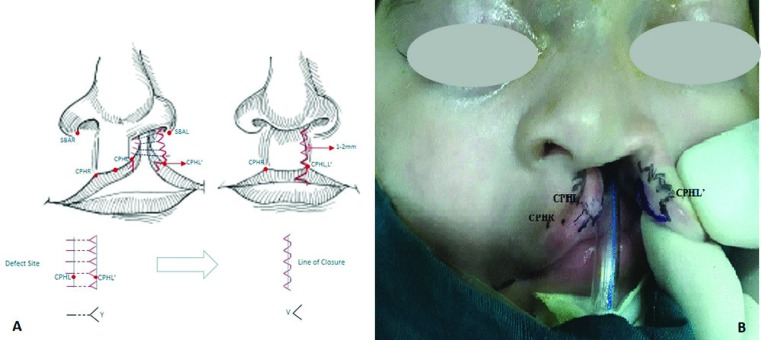
**A:** Schematic view of operation, **B:** Design and lip markings, (Noordhoft’s points). SBAR: Sub-Alar- Right. SBAL: Sub- Alar- Left. CPHR: Crista Philtri – Right .CPHL: Crista- Philtri –Left. CPHL׳: Crista - Philtri -Left (cleft side)

Regarding the design and lip markings, success in cleft lip repair is equally dependent on the repair design and technical skill. In order to repair unilateral cleft lip repair, crista philtri right’ (cphr’), crista philtri left’ (cphl’) points (Noordhoft’s points)^4^ were determined (Figure 1). The first arm of Y (head of Y) in subalare (sbal) area (Figure 1) and the second and third arms were drawn 1-2 mm away from the designed Y in alar side of the cleft (Figure 1). In this method, we avoided extending the incision through the alar crease of the alar side except in overwhelming cleft with extreme lateral displacement of the alar base. 

Depending on the severity of discrepancy and width of the cleft, there might also be a need for the design of the fourth Y flap to create sufficient lip height. In severe cases, it was necessary to move cphl’ point a little laterally and make the V part of the Y arm deeper on the lateral side to maintain sufficient lip height for the cleft philtral side. Next the separated Y flap in the dry mucosa was designed at a distance between the cphl’ and wet‒dry junction (Figure 1). The third arms of the lower and last Y flap in the upper part of the vermillion matched the famous triangular flap.

**Fig. 2:**
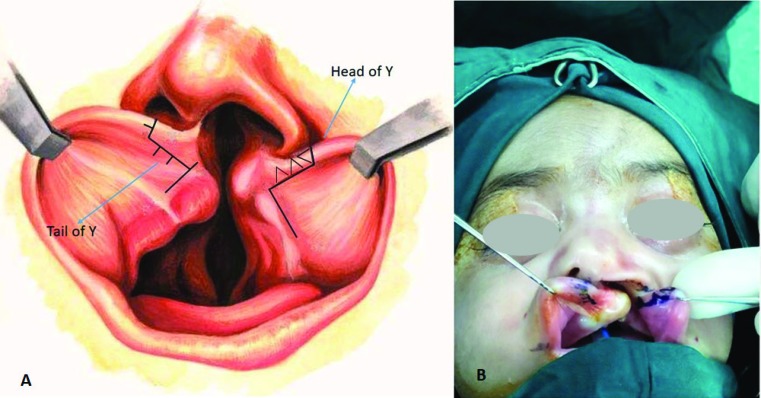
Separated Y flap in the wet mucosa to increase the length of closure line in wet mucosa. **A:** schematic view, **B:** intraoral markings

The last separated Y flap in the wet mucosa had one to three arms to increase the length of closure line in wet mucosa (Figure 2). Also mucosal excess in wet mucosa might be due to the hypertrophic scar arising from the overpowering tension and insufficient wound length. Therefore, by increasing the length of the wound in wet mucosa, the incidence of mucosal excess known as an unfavorable complication of unilateral cleft lip repair decreased.

For unilateral cleft lip repair, operative technique with separated Y-to-V flaps under magnification was used. We preferred to inject no solutions to prevent any changes at critical connection points. At the end of the operation, to decrease postoperative pain, 1-2 mL of lidocaine containing epinephrine at a concentration of 1:200000 was injected into both sides of the lip. Since malposition of the landmarks would lead to an asymmetrical lip and unsatisfactory aesthetic results, we used Zeiss Vario 700 microscope in this technique. The incision was marked as a maximal curvilinear line from cphl extending upward to the lateral base of the columella and back cut to the nasolabial junction if needed. On the cleft side, incision of multiple arms of separated Y was carried out from the alar base of the cleft side to the top of the vermilion.

After designing the separated Y flaps in dry and wet mucosa, the incision was continued from this area to the sulcus region. Usually in the case of incomplete unilateral cleft lip, and there was no need for double-sided cutting in the sulcus. In the case of severe and complete unilateral cleft lip through double-sided sulcus incisions in the direction of maxillary arch on two sides of the cleft, complete lip dissection was made in subperiosteal plane. Lateral side dissections was continued to the outside of the infraorbital nerve, and on the medial side to be continued to the frenulum and columella base. In the case of complete unilateral cleft lip, in the first stage of surgery unilateral vomer and partial inferior turbinate flap was used to close the hard palate, alveolar ridge and nasal floor according to a previous study.^15^

The muscle layers were dissected along the skin boarder. Abnormal muscle insertion beneath the columella, alar base and nasal floor was adequately released as described before.^16^ Muscle cuff was completely released from the skin mucosa and lip border bilaterally through the alar base on the cleft side and midline philtrum on the non-cleft side based on previous reports.^17^^,^^18^ Closure was started at wet-dry junction as a key point and then, the mucosal layer was closed from the sulcus up to this point. Then the muscles were approximated in U mattress and the overlapping muscles were approximated as described in the literature to achieve an elevation from the columella to the vermillion for imitation of the cleft side philtral column.^17^^,^^18^

**Fig. 3:**
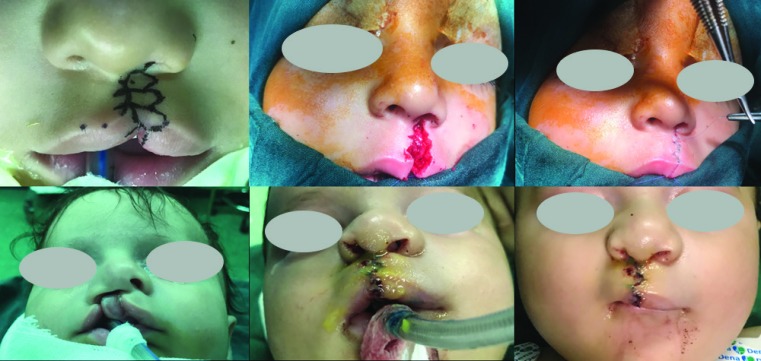
**A:** Multiple Y to V flap design, **B:** tension apply in the wet-dry junction suture about 1-2 mm in overcorrected position versus the non-cleft philtral side, **C:** Final closure. (Case 1). **D:** wide unilateral cleft lip and palate (pre op), **E:** correction the position of the lip and nose, **F:** Final closure (Case 2)

The uppermost muscular stitch in most cases was sutured to the caudal area of the nasal septum, just above the anterior nasal spine with 4/0 ethilon or polydioxanone (PDS) sutures. For simultaneous correction of the cleft lip and nose deformity at the time of labial rearrangement, we used the combination techniques described before.^19^^-^^24^ After completion of the closure of the muscular layer from the top to bottom and treatment of cleft lip nose deformity, skin closure was carried out as the most important and unique part of our technique. First, we applied tension in the wet-dry junction suture about 1-2 mm in overcorrected position versus the non-cleft philtral side (Figure 3).

Then the tail of separated Y was cut on the cleft side philtrum just in front of the Y head. Depending on the severity of the unilateral cleft lip, depth of the Y head, Y tail was cut on the philtral side. We avoided extending the incision through the midline and in most cases limited it to the new philtral column. Skin closure was started at white skin roll with 7/0 Ethilon sutures. Then Y to V was completed with integration of head of Y to the Y tail up to the nostril sill. In this step, small parts of tissues that interrupted interdigitation of Y to V flaps were removed.

## RESULTS

This technique was based on the principles of plastic surgery and the absence of a straight-line closure along the skin and mucosa for the repair the unilateral cleft lip. This issue is of utmost importance in cases with severe loss of lip height and discrepancy. In most cases of unilateral cleft lip on the non-philtral edge there was excess tissue and on the philtral side, there was tissue shortage. The maximum soft tissue was available on the non-philtral edge to increase the height of the lip on the philtral side. Regarding beauty and performance, multiple Y-to-V flaps provided the best results (Figure 3).

## DISCUSSION

Similar to Fisher and Sommerlad (2011), in complete unilateral cleft lip in the first stage of surgery, unilateral vomer and partial inferior turbinate flap were used for closure of the hard palate, alveolar ridge and nasal floor according to a previous study.^15^ The muscle layers were identically dissected along the skin boarder and abnormal muscle insertion below the columella, alar base and nasal floor was adequately released as described by Raymond (2012).^16^ Chen et al. (2018) and and Travis (2016) reported muscle cuff to be completely released from the skin mucosa and lip border to be bilaterally through the alar base on the cleft side and midline philtrum on the non-cleft side which are in line with our findings.^17^^,^^18^

They have also shown that closure was started at wet-dry junction and the mucosal layer was closed from the sulcus and the muscles were approximated in U mattress and the overlapping muscles were approximated to achieve an elevation from the columella to the vermillion for imitation of the cleft side philtral column.^17^^,^^18^ Similar to our results, several authors reported that the uppermost muscular stitch has been sutured to the caudal area of the nasal septum, above the anterior nasal spine and for simultaneous correction of the cleft lip and nose deformity at the time of labial rearrangement, the combination techniques were used.^19^^-^^24^

We believe that with separated Y-to-V-plasty under magnification many problems regarding unilateral cleft lip, especially in severe defects are solved with no need for further surgery and revision. However, the effects of this method on maxillary growth, cleft lip and nose deformity and long-term outcomes are unclear and further studies are necessary. Therefore, we recommend use of the novel separated Y-to-V-plasty to achieve a natural and balanced shape in unilateral cleft lip repair. We have come to the conclusion, by experience, that the best results in term of beauty and performance are achieved when multiple Y-to-V flaps are used. By designing more flaps, we can reduce the depth of Y-to-V arms and achieve higher lip height despite the small width of the wound.

## CONFLICT OF INTEREST

The authors declare no conflict of interest.
